# Neoadjuvant chemotherapy with Cisplatin in BRCA1 mutation carriers – results of treatment

**DOI:** 10.1186/1897-4287-10-S3-A3

**Published:** 2012-04-20

**Authors:** T Byrski, J Gronwald, T Huzarski, E Marczyk, P Blecharz, O Ashuryk, C Cybulski, D Zuziak, R Wiśniowski, D Godlewski, SA Narod, J Lubiński

**Affiliations:** 1 Pomeranian Medical University, Clinic of Oncology, Szczecin, Poland; 2 Oncology Institute, Kraków, Poland; 3 Regional Oncology Center, Bielsko-Biała, Poland; 4 Center for Epidemiology and Prevention, Poznań, Poland; 5 Women’s College Research Institute, Toronto, Ontario, Canada

## Aim of study

The aim of this study was to evaluate the frequency of pathological complete response (pCR) in BRCA1 mutation carriers diagnosed with breast cancer treated with preoperative Cisplatin chemotherapy.

## Material and methods

Between December 2006 and August 2011 seventy five women with BRCA1 mutation and diagnosed with breast cancer stage I to III were enrolled. Patients were treated with Cisplatin at dose 75 mg/m2 every three weeks for four cycles. After chemotherapy mastectomy was performed and followed with conventional chemotherapy. Seven patients had prior chemotherapy for a previous cancer diagnosis. Four patients received prior chemotherapy for their current cancer diagnosis and then received therapy according to our protocol.

In the group of 67 patients treated with Cisplatin in first line of treatment - 32 met the standard criteria for neoadjuvant chemotherapy, and in 35 cases - the criteria were not met.

Pathologic complete response was determined by review of surgical specimens. Complete pathologic response was defined as no residual invasive disease in both the breast and axilla. Information was collected on clinical stage, grade, hormone receptor status and HER2 status prior to treatment.

## Results

A pathologic complete response (pCR) was observed in 47/75 (62,7%) patients, partial response was observed in 27/75 (36%) patients and stable disease in 1/75 (1,3%) patient. After excluding those patients who were treated with chemotherapy in the past - pathological complete response was achieved in 45/67 (67,2%) patients. In the group of 25 patients who met the standard criteria for neoadjuvant chemotherapy and treated with Cisplatin-monotherapy as a first line of treatment pathological complete response was achieved in 56% (14/25) patients. In group of remaining 42 patients who did not meet the criteria for neoadjuvant treatment the pathological complete response was achieved in 78,6% (33/42) patients.

